# Nonspecific Symptoms Attributable to Lyme Disease in High-Incidence Areas, United States, 2017–2021

**DOI:** 10.3201/eid3114.250459

**Published:** 2025-12

**Authors:** Courtney C. Nawrocki, Mark J. Delorey, Austin R. Earley, Sarah A. Hook, Kiersten J. Kugeler, Grace E. Marx, Paul S. Mead, Alison F. Hinckley

**Affiliations:** Centers for Disease Control and Prevention, Fort Collins, Colorado, USA (C.C. Nawrocki, M.J. Delorey, A.R. Earley, S.A. Hook, K.J. Kugeler, G.E. Marx, P.S. Mead, A.F. Hinckley); Oak Ridge Institute for Science and Education, Oak Ridge, Tennessee, USA (A.R. Earley)

**Keywords:** Lyme disease, *Borrelia*, post-treatment Lyme disease syndrome, vector-borne infections, bacteria, cognitive impairment, fatigue, pain, United States

## Abstract

For some patients who have Lyme disease (LD), nonspecific symptoms can persist after treatment and impair quality of life. Estimating the frequency and duration of such symptoms is challenging. Using commercial insurance claims data from 2017–2021 for enrollees residing in states where LD is common, we identified 24,503 case-patients with LD and matched them (1:5) with 122,095 control-patients with other diagnoses by demographics, medical service date, and inpatient/outpatient setting. We compared relative frequencies of diagnosis codes for pain, fatigue, and cognitive difficulties between case-patients and control-patients in the year after diagnosis. Those symptom codes occurred 5.0% more frequently among case-patients than among control-patients and comprised »11.0% of the total symptom codes among case-patients. Symptom code frequency among case-patients declined significantly in the 6–12 months after LD diagnosis and reached levels similar to control-patients by the end of the year, with the exception of fatigue.

Lyme disease (LD) is a tickborne illness caused in North America by the bacteria *Borrelia burgdorferi* and *B. mayonii*. Human cases occur primarily in the northeastern and upper midwestern United States (*1*). Most patients recover completely when treated with appropriate antimicrobial drugs (*2*–*4*); however, some report prolonged nonspecific symptoms of pain, fatigue, or cognitive difficulties (*5*–*12*). Those prolonged symptoms are often referred to as post-treatment Lyme disease syndrome (PTLDS) and can occur in the absence of objective chronic sequelae such as facial palsy or recurrent arthritis. Persistence of similar nonspecific symptoms has been reported after other infections, including COVID-19, which suggests a common mechanism underlying such infection-associated chronic conditions and illnesses (*13*).

Published studies describing the frequency and duration of nonspecific symptoms after acute LD have several limitations. Some have lacked a control group. Because symptoms of pain, fatigue, and cognitive difficulties are commonly experienced by the general population, inclusion of a control group is essential to determine the fraction of symptoms specifically attributable to LD. Other studies have been challenging to contextualize because of variations in methodology, patient groups, and timing of assessment (*4*,*14*–*18*). Among recent studies that are methodologically similar and include controls, 5 have reported on the frequency of nonspecific symptoms at 6 and 12 months after treatment for patients with early localized LD (i.e., erythema migrans rash). Two studies (*16*,*18*) reported elevated frequencies 6 months after treatment for >2 symptom types among case-patients compared with control-patients; 3 other studies reported no notable differences in relative frequencies for the symptom types of pain, fatigue, or cognitive difficulties (*11*,*14*,*19*). One of the 5 studies reported significantly elevated symptom frequencies among case-patients at 12 months posttreatment (*16*). Nevertheless, most of those recent studies identified a small subset of patients having prolonged symptoms consistent with PTLDS during 12 months of follow-up (*11*,*16*,*18*).

Large health record databases, such as those containing electronic health records or insurance claims records, have been used to identify and evaluate LD diagnoses, including the frequency of nonspecific symptom diagnosis codes suggestive of PTLDS in the year after LD diagnosis (*20*,*21*). In this study, we used a large insurance claims database to determine the frequency and risk for nonspecific symptom codes suggestive of PTLDS that were attributable to LD during the 12 months after diagnosis. 

## Methods

### Data Source

In this matched cohort study, we used 2017–2021 data from the Merative MarketScan Commercial Claims and Encounters Databases (*22*), which contains annual insurance claims information for >25 million US residents <65 years of age with employer-sponsored health insurance and their dependents. We restricted the eligible patient population for this study to those who resided in states with a high incidence of LD (defined as >10 confirmed cases of LD per 100,000 population for 3 years) (*23*). Centers for Disease Control and Prevention human subjects review determined that this project did not involve human subjects. Thus, Institutional Review Board approval was not required.

### Identification of LD Case-Patients

To identify LD case-patients, we used a previously developed algorithm (*23*) based on International Classification of Diseases, 10th Revision, Clinical Modification (ICD-10-CM), codes for LD (A69.2x) and a prescription claim for >7 days of treatment with a recommended first-line drug for LD within 14 days before or after the date of the the ICD-10-CM code. We identified inpatient diagnoses solely on the basis of whether an ICD-10-CM code for LD was listed as the primary or secondary diagnosis code.

We excluded case-patients with <365 days of continuous health plan enrollment immediately before and after their LD diagnosis date. To increase the probability that we included only new LD diagnoses, we also excluded patients with an LD ICD-10-CM code in the 365 days before they met LD case-patient criteria. Patients could meet LD diagnosis criteria multiple times in the 5-year study period, but we included only the first instance per calendar year.

### Selection of Matched Control-Patients

We identified a 5% random sample of all eligible MarketScan enrollees each year during the 5-year study period. We then matched the potential control-patients individually to case-patients without replacement on age group (0–17, 18–34, 35–44, 45–54, 55–64 years), sex, and inpatient versus outpatient diagnosis. Of the potential control-patients meeting the matching criteria for a case-patient, we considered for selection only those having a healthcare visit within +14 days of the case-patient’s LD diagnosis date. All potential control-patients had to have >365 days of continuous enrollment immediately before and after their matched date. We required >1 control-patient per case-patient to a maximum of 5. Persons who met case-patient criteria in a given year were ineligible to be control-patients in that year.

### Identification of Nonspecific Symptom Codes

We identified nonspecific symptoms suggestive of PTLDS by specific healthcare encounter-associated ICD-10-CM diagnosis codes in case-patient and control-patient claims occurring 365 days before to 365 days after the matched diagnosis date. We noted such symptoms in the categories of pain, fatigue, and cognitive difficulties (Appendix Table 1).

**Table 1:** Distribution of characteristics of among Lyme disease case-patients and matched control-patients in in study of nonspecific symptoms attributable to Lyme disease in high-incidence areas, United States, 2017–2021*

Characteristic	No. (%)	
	Case-patients, n = 24,503†	Control-patients, n = 122,095
Year		
2017	6,550 (22.7)	32,632 (22.7)
2018	5,257 (23.2)	26,140 (23.1)
2019	5,303 (22.5)	26,472 (22.5)
2020	3,668 (17.5)	18,267 (17.5)
2021	3,725 (14.2)	18,584 (14.2)
Age group, y		
0–17	6,101 (23.7)	30,681 (23.8)
18–34	3,587 (22.5)	18,170 (22.8)
35–44	3,398 (15.0)	17,075 (15.1)
45–54	5,582 (18.9)	27,671 (18.8)
55–64	5,835 (19.9)	28,498 (19.5)
Sex		
F	11,088 (44.5)	55,316 (44.6)
M	13,415 (55.5)	66,779 (55.4)
Season of onset		
Winter, Dec–Feb	1,670 (6.9)	8,311 (6.9)
Spring, Mar–May	4,316 (17.4)	21,498 (17.4)
Summer, Jun–Aug	13,704 (56.1)	68,307 (56.1)
Fall, Sep–Nov	4,813 (19.6)	23,979 (19.6)
Diagnosis encounter type		
Outpatient	24,241 (98.9)	120,978 (99.1)
Inpatient	262 (1.1)	1,117 (0.9)

*Case-patients and control-patients were matched on age group, sex, Lyme disease diagnosis or healthcare visit date, and inpatient vs. outpatient status. †Unweighted frequencies reflect distributions of variables in the sample; weighted percents reflect distributions of variables in MarketScan database.

### Analysis

We calculated weighting for each observation (Appendix) and incorporated it into all point and variance estimates. We compared the weighted proportion of symptom codes among case-patients and control-patients by month and symptom category (pain, fatigue, cognitive difficulties) in the 2–12 months (hereafter referred to as the year) before and after diagnosis. The 30 days before and after the diagnosis date were the wash-out period, in which we considered any symptom codes to be likely attributable to acute illness rather than persistent symptoms. To evaluate associations between having a diagnosis of LD and nonspecific symptom codes in the year before and after diagnosis, we calculated risk ratios (RRs) and attributable risk percents (i.e., the percentage of incidence of disease in exposed persons that is a result of the exposure), as well as 95% CIs around differences and ratios. We report observed frequencies but weighted proportions throughout. We extracted data using SAS version 9.4 (SAS Institute Inc., https://www.sas.com) and performed analyses in R version 4.4.0 or 4.4.1 (The R Project for Statistical Computing, https://www.r-project.org).

## Results

A total of 24,503 LD diagnoses (24,197 unique persons) met case-patient criteria during 2017–2021. The highest number of case-patients was 6,550 in 2017 and the lowest was 3,668 in 2020 (Table 1). Nearly all (99.1%) case-patients had 5 matched control-patients.

### Nonspecific Symptom Prevalence and Risk Ratios in the Postdiagnosis Year 

**Figure 1:**
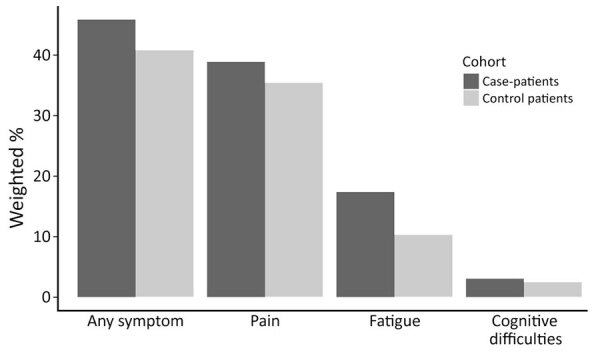
Weighted percentages of case-patients and control-patients with nonspecific symptom codes from the International Classification of Diseases, 10th Revision, Clinical Modification, in the year postdiagnosis excluding wash-out period in study of nonspecific symptoms attributable to Lyme disease in high-incidence areas, United States.

Approximately 46% of case-patients and 41% of control-patients had >1 diagnosis code for any nonspecific symptoms related to pain, fatigue, or cognitive difficulties in the postdiagnosis year, representing an absolute difference of 5% (Figure 1). Upon calculating attributable risk percent, an estimated 11.0% of symptom codes among case-patients were a result of their LD diagnosis (Table 2). Pain was the most common symptom code category among both groups, followed by fatigue, then cognitive difficulties. Although less common than pain, fatigue was the symptom code category with the highest relative risk for case-patients compared with control-patients in the postdiagnosis year (RR = 1.67 [95% CI 1.61–1.72]) (Table 2).

**Table 2:** Risk for nonspecific symptom codes among case-patients and control-patients in the prediagnosis and postdiagnosis years in study of nonspecific symptoms attributable to Lyme disease in high-incidence areas, United States, 2017–2021

Measure	Any symptom	Pain	Fatigue	Cognitive difficulties
Risk ratio prediagnosis (95% CI)	1.05 (1.03–1.07)	1.03 (1.01–1.05)	1.35 (1.29–1.41)	0.91 (0.81–1.01)
Risk ratio postdiagnosis (95% CI)	1.12 (1.11–1.14)	1.10 (1.08–1.12)	1.67 (1.61–1.72)	1.18 (1.08–1.28)
Ratio of relative risks (95% CI)	1.07 (1.05–1.10)	1.07 (1.04–1.10)	1.24 (1.17–1.30)	1.30 (1.13–1.49)
Attributable risk postdiagnosis, %	10.9	8.7	40.2	16.1

*Symptom codes from the International Classification of Diseases, 10th Revision, Clinical Modification.

### Changes in Relative Frequency of Nonspecific Symptoms during the Postdiagnosis Year 

**Figure 2:**
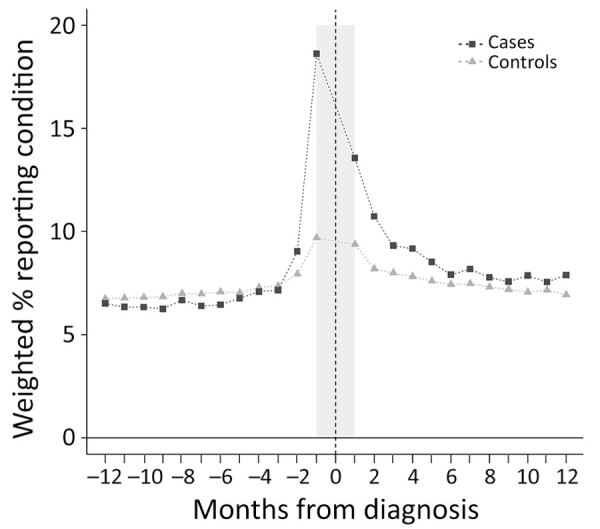
Weighted percentages of case-patients and control-patients with any nonspecific symptom code from the International Classification of Diseases, 10th Revision, Clinical Modification, by month in the year prediagnosis and postdiagnosis, in study of nonspecific symptoms attributable to Lyme disease in high-incidence areas, United States.

**Figure 3:**
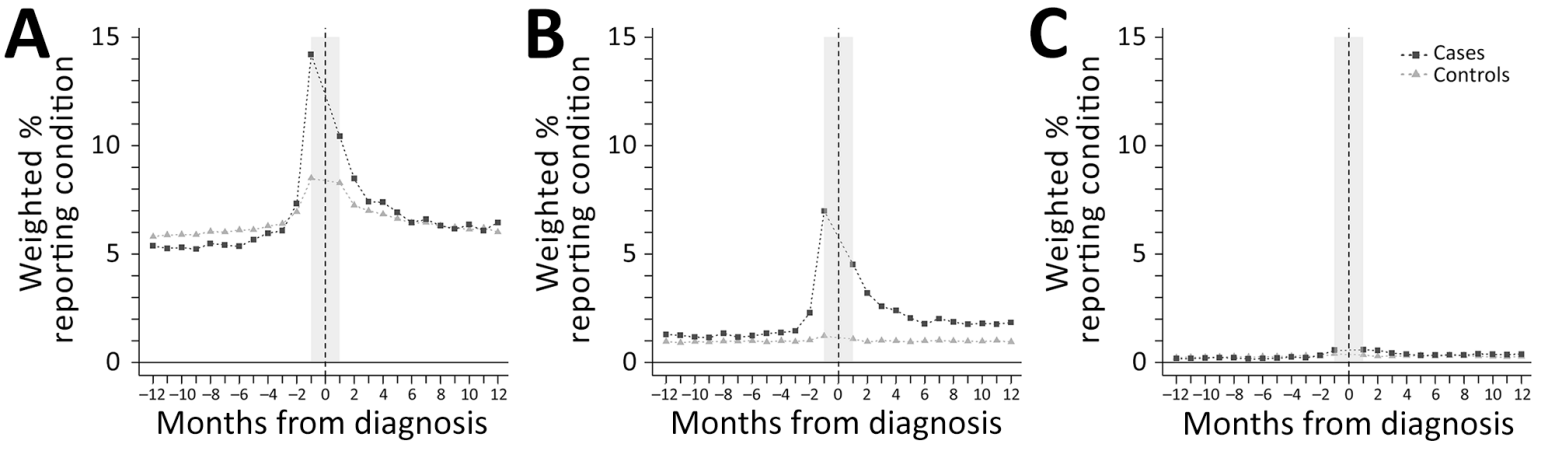
Weighted percentages of case-patients and control-patients with codes from the International Classification of Diseases, 10th Revision, Clinical Modification, in each nonspecific-symptom category, by month in the year prediagnosis and postdiagnosis, in study of nonspecific symptoms attributable to Lyme disease in high-incidence areas, United States. A) Pain; B) fatigue; C) cognitive difficulties.

The relative frequency of any symptom code among case-patients declined statistically during the year after LD diagnosis, becoming similar to control-patients by the end of the year (Figure 2). The average percent of excess symptom codes among case-patients compared with control-patients declined from 2.5% in the 2nd month to 0.5% in the 6th month (difference = 2.1% [95% CI 1.5%–2.6%) and 1.0% in the 12th month (difference = 1.6% [95% CI 1.0%–2.2%]) postdiagnosis (Table 3). Of the 3 symptom categories, pain declined most precipitously in relative frequency among case-patients, becoming similar to that of control-patients at ≈6 months (Figure 3, Table 3). Similarly, we observed fatigue symptom codes more often among case-patients than control-patients in the first 6 months postdiagnosis; however, relative frequency among case-patients stabilized and remained slightly elevated (≈1.0%) over that reported for control-patients during the remaining 6 months of the postdiagnosis year. The relative frequency of codes for cognitive difficulties was extremely low (<0.1%) for both case-patients and control-patients and varied little in the postdiagnosis year.

**Table 3:** Average percentage difference in relative frequency of nonspecific symptoms between Lyme disease case-patients and control-patients over the postdiagnosis year in study of nonspecific symptoms attributable to Lyme disease in high-incidence areas, United States, 2017–2021

Symptom	2 mo postdiagnosis	6 mo postdiagnosis	12 mo postdiagnosis	% Difference (95% CI) between 2 and 12 mo	% Difference (95% CI) between 6 and 12 mo
Any symptom	2.5	0.5	1.0	1.6 (1.0–2.2)	−0.5 (−1.0 to 0.06)
Pain	1.2	0.0	0.4	0.8 (0.3–1.3)	−0.4 (−1.0 to −0.1)
Fatigue	2.3	0.8	0.9	1.4 (1.1–1.7)	−0.1 (−0.4 to 0.2)
Cognitive difficulties	0.3	0.0	0.1	0.2 (0.0–0.3)	−0.06 (−0.2 to 0.1)

### Comparing Symptom Prevalence and Risk Ratios in the Prediagnosis Versus Postdiagnosis Year 

Approximately 33% of case-patients and 32% of control-patients had >1 code for a nonspecific symptom in the same category in both the year before and year after diagnosis date. When we evaluated by specific symptom category, we found proportions of symptom codes in the prediagnosis year were similar for case-patients and control-patients for all conditions. When we compared the relative risk for symptom codes in the prediagnosis year to the relative risk for symptom codes in the postdiagnosis year (Table 2), the relative risk for pain among case-patients was 1.07 times as high in the postdiagnosis year (95% CI 1.04–1.10), fatigue was 1.24 times as high (95% CI 1.17–1.30), and cognitive difficulties was 1.30 times as high (95% CI 1.13–1.49).

## Discussion

In this investigation of a large commercial insurance claims dataset, we observed that 5% more LD case-patients had a code for a nonspecific symptom in the categories of pain, fatigue, or cognitive difficulties than did control-patients in the year after their diagnosis. The relative risk of experiencing nonspecific symptoms at any point in the postdiagnosis year was statistically higher for case-patients (Table 2) but varied substantially based on postdiagnosis month, symptom category, and whether those symptom category codes also occurred in the year before LD diagnosis. The frequency of symptom codes among case-patients declined statistically over the 6 months after LD diagnosis and treatment (Table 3); codes for pain and cognitive difficulties reached proportions that were not statistically different from those of control-patients by the end of the postdiagnosis year. Although the frequency of fatigue codes also diminished significantly (Table 3; Figure 3) over time among case-patients, it was still slightly elevated (≈1%) compared to controls at 12 months postdiagnosis among LD case-patients. Our findings are consistent with several previous clinical studies (*3*,*11*,*18*) identifying similar persisting symptoms of unclear pathogenesis among a subset of persons who received diagnosis and treatment for LD.

Symptoms of pain, fatigue, and cognitive difficulties are common in the general population and have many causes. We observed a 5% excess of nonspecific symptom codes among case-patients amid an overall high background prevalence of those same codes. On that basis, we calculated that ≈11% of those nonspecific symptoms experienced by case-patients were attributable to having had LD, meaning that 89% were likely from other causes. That situation might explain some of the difficulty in identifying effective treatments for PTLDS (*24*–*27*). In addition, >30% of both case-patients and control-patients had >1 of these symptom category codes in the year before diagnosis. For case-patients, it is possible that some of those preexisting codes represented symptoms indicative of LD that were not recognized, diagnosed, or treated until a later clinical visit (our assigned diagnosis date). Studies have consistently reported on higher rates of prolonged symptoms among patients with disseminated manifestations or longer durations of disease before effective treatment (*16*,*28*–*32*). However, severe fatigue, cognitive impairment, or pain before diagnosis has also been shown to be a determinant of persistent symptoms after LD diagnosis and treatment. In a previous study (*33*), the main predictors of persistent symptoms were lower social and physical functioning, negative illness perceptions, and anxiety and depression, factors that we could not assess in this claims-based study. It is also possible that these codes might have been assigned, both before and after the diagnosis date, for symptoms unrelated to a LD diagnosis.

The excess frequency of nonspecific symptom codes observed for case-patients is similar to that reported in 2021 in the largest prospective study of clinically confirmed LD patients published as of March 2025 (*16*), which reported a prevalence of persistent symptoms that was 3.9%–6.0% higher than that of controls over the postdiagnosis year. That study actively and systematically collected information on occurrence of all symptoms at regular intervals from all study participants and further identified participants who had symptoms that began within 6 months of a LD diagnosis and persisted for >6 months. Although our study involved data collected for billing purposes and thus our methodology is notably different from theirs, we expect that both the persistent-symptoms group from Ursinus et al. (*16*) and the healthcare-seeking patients in our study represent persons with more severe or unusual symptoms. Ursinus et al. (*16*) also observed a higher proportion of patients with disseminated LD (e.g., Lyme arthritis or cranial neuritis) experienced persistent symptoms of fatigue and pain compared with those with early localized disease (i.e., erythema migrans rash); resolution of symptoms over time occurred primarily for the patient group having disseminated manifestations. Given the limitations of claims data analyses, we were not able to evaluate in our study the effects of specific LD manifestations.

The excess 5% of nonspecific symptoms observed for case-patients in our study was somewhat lower than that reported in other recent evaluations of large health databases. Although we used methodology similar to that of Moon et al. (*21*), those authors found a 9% difference between case-patients and control-patients for symptoms occurring anytime in the postdiagnosis year in an evaluation of electronic health records from a Pennsylvania health system. The diagnosis codes we used to identify nonspecific symptoms were converted from the ICD-9-CM codes used in that previous study (*21*) to ICD-10-CM, because ICD-9-CM codes were phased out and replaced by ICD-10-CM codes in 2015. Although it is possible that we missed some relevant codes in the conversion and thus did not include them in our study, our symptom code list was also somewhat broader; we included additional codes for specific joint and limb pain, consistent with other past clinical studies of subjective symptoms after LD (*31*,*34*). Last, our estimate is much lower than the 35.3% difference in relative frequency of symptom codes occurring over the postdiagnosis year between case-patients and control-patients as reported previously (*20*). The primary difference between that study and ours was that the previous study (*20*) included codes during the 1 month immediately after LD diagnosis, whereas we did not include codes during that period because they would likely represent symptoms associated with acute LD.

The first limitation of this study is that we relied on observational claims data for information on patient care-seeking and were unable to verify those data by medical record review. We interpreted ICD-10-CM codes as provider diagnoses, but such codes are assigned primarily for billing purposes and thus may not accurately reflect actual diagnosis or the reason for seeking care. In addition, ICD-10-CM codes are subject to varying provider coding practices, leading to the potential for both overestimation and underestimation of the true prevalence of nonspecific symptoms among our study population and potential misclassification of LD case-patients and control-patients. Second, we were unable to assess symptom severity. Clinical studies have found that greater symptom severity at time of LD treatment increases risk of experiencing prolonged symptoms (*8*,*35*). Although information on severity is not available in claims data, we might expect that most of the codes recorded in claims were the result of complaints that warranted care-seeking and were not those experienced more regularly by a substantial portion of the population (*36*). Nevertheless, ability to assess symptom severity would have lent additional confidence to the identification of potential PTLDS symptoms and provided the opportunity to evaluate qualitative improvement of symptoms over time. Third, we did not evaluate LD case-patients for potential co-infections or underlying conditions that could have caused prolonged symptoms, leading to possible overestimation of LD-associated symptoms among case-patients. However, in a sensitivity analysis in which we removed case-patients with diagnosis codes for pain, fatigue, or cognitive difficulties in the year before diagnosis to control for preexisting conditions, we found minimal changes to observed symptom trends (Appendix Figures 1, 2). Fourth, we conducted this analysis among residents of high-incidence states, where healthcare providers are more experienced with diagnosing LD. The relative frequency of symptoms after LD diagnoses in emerging or low-incidence areas might be different because of variation in ascertainment or coding practices. Fifth, although a very large convenience sample, MarketScan lacks data on persons who are uninsured, >65 years of age, or military personnel, and it is not nationally or otherwise representative. Last, the observed decrease in LD case-patients during the study period coincides with an overall decrease in MarketScan enrollees during that time because of changing data contributors. On the basis of past evaluations of LD diagnoses in MarketScan (*37*) and our use of weighting, we do not expect that the decrease affected our results.

Certain biases may have also affected our study results. Misinformation, coupled with limited diagnostic testing, has contributed to confusion and controversy about LD (*38*,*39*). It is possible that observed patterns were influenced by patient or healthcare provider beliefs about the disease. For example, some patients with a recent LD diagnosis might expect to have long-term, nonspecific symptoms and would therefore be more likely to report or seek healthcare for those symptoms. Similarly, healthcare providers might be more likely to ask about or record symptoms for patients having had LD (*40**,**41*). A second potential bias is our selection of controls from among a care-seeking population that could potentially overrepresent generally sicker persons (*21*,*42*). If that is the case for those claims data, we might have overestimated the baseline frequency of pain, fatigue, and cognitive difficulties and underestimated the proportion of symptoms attributable to LD diagnoses.

Ultimately, this analysis of a large commercial claims dataset supports observations made in past clinical and epidemiologic studies that a minority of patients with LD diagnosis will experience symptoms of pain, fatigue, or cognitive difficulties for months beyond the acute illness period. Although it appears that most of those symptoms will improve or resolve in the 6 months after diagnosis, a smaller subset of patients might continue to experience persistent symptoms that affect their daily quality of life. More studies are needed to understand the underlying factors associated with occurrence and persistence of such symptoms to inform appropriate treatment and care. Until more is known, guidance on caring for patients with clinically similar infection-associated chronic conditions and illnesses, such as long COVID, will be useful.

Appendix Additional information from study of nonspecific symptoms attributable to Lyme disease in high-incidence areas, United States.
