# Supplementary material for: Nonspecific Symptoms Attributable to Lyme Disease in High-Incidence Areas, United States, 2017–2021
Source: Emerg Infect Dis. 2025 Dec;31(Suppl 2):S30–7. doi: 10.3201/eid3114.250459 (PMC12829481; doi:10.3201/eid3114.250459)
Supplement: Appendix — Additional information from study of nonspecific symptoms attributable to Lyme disease in high-incidence areas, United States. [file 25-0459-Techapp-s1.pdf]

Article DOI: https://doi.org/10.3201/eid3114.250459

EID cannot ensure accessibility for supplementary materials supplied by authors. Readers who have difficulty accessing supplementary content should contact the authors for assistance.

Nonspecific Symptoms Attributable to Lyme Disease in High-Incidence Areas, United States, 2017–2021

Appendix

Additional Information on Statistical Methodology

We used a stratified design, with matched groups as the strata. Weighting for each observation was calculated by raking according to the marginal distributions of sex, age group, and year in the MarketScan database during 2017–2021.

Intervals for differences were computed with weighted standard errors and t-distribution critical value with degrees of freedom based on survey design. Intervals for ratios were computed according to Zou and Donner (*1*).

Sensitivity Analysis of the Prevalence of Nonspecific Symptom Codes among Case-Patients and Control-Patients with New Onset Symptoms

We analyzed the difference in proportions among the subset of case-patients and control-patients with evidence of "new onset" symptoms, defined as those occurring for the first time in the year postdiagnosis and not during the year before diagnosis. Overall, 27% of case-patients and 20% of control-patients had a new onset symptom code in the year postdiagnosis, meaning no code was present in that specific symptom type category at any point before the wash-out period. Temporal trends in relative frequencies of symptom codes for the subset with new onset symptom codes were generally similar to those observed overall in the year postdiagnosis (Appendix Figure 1).

Appendix Table. International Classification of Diseases, 10th Revision, Clinical Modification (ICD-10-CM) codes for nonspecific symptoms suggestive of post-treatment Lyme disease syndrome

Symptom category	ICD-10-CM code	Description
Pain	G43x*	Migraine
	G44x*	Headache syndromes
	G50.1	Atypical face pain
	G89.29	Other chronic pain
	G89.4	Chronic pain syndrome
	M25.5x*	Pain in joint
	M60.9	Myositis, unspecified
	M79.1	Myalgia
	M79.6x*	Pain in limb
	M79.7	Fibromyalgia
	R51.9	Headache, unspecified
	R52	Pain, unspecified
Fatigue	G93.3x*	Postviral and related fatigue syndromes
	M62.81	Muscle weakness (generalized)
	R53.1	Weakness
	R53.8x*	Other malaise and fatigue
Cognitive difficulties	F03x*	Unspecified dementia
	F04x*	Amnestic disorder due to known physiologic condition
	F06.7x*	Mild neurocognitive disorder due to known physiologic condition
	F06.8	Other specified mental disorders due to known physiologic condition
	G93.40	Encephalopathy, unspecified
	G93.49	Other encephalopathy
	G31.84	Mild cognitive impairment of uncertain or unknown etiology
	R41.0	Disorientation, unspecified
	R41.1	Anterograde amnesia
	R41.2	Retrograde amnesia
	R41.3	Other amnesia
	R41.82	Altered mental status, unspecified
	R41.83	Borderline intellectual functioning
	R41.840	Attention and concentration deficit
	R41.844	Frontal lobe and executive function deficit
	R41.89	Other symptoms and signs involving cognitive functions and awareness
	R41.9	Unspecified symptoms and signs involving cognitive functions and awareness

*“x” indicates that all subsequent sub-codes were included.

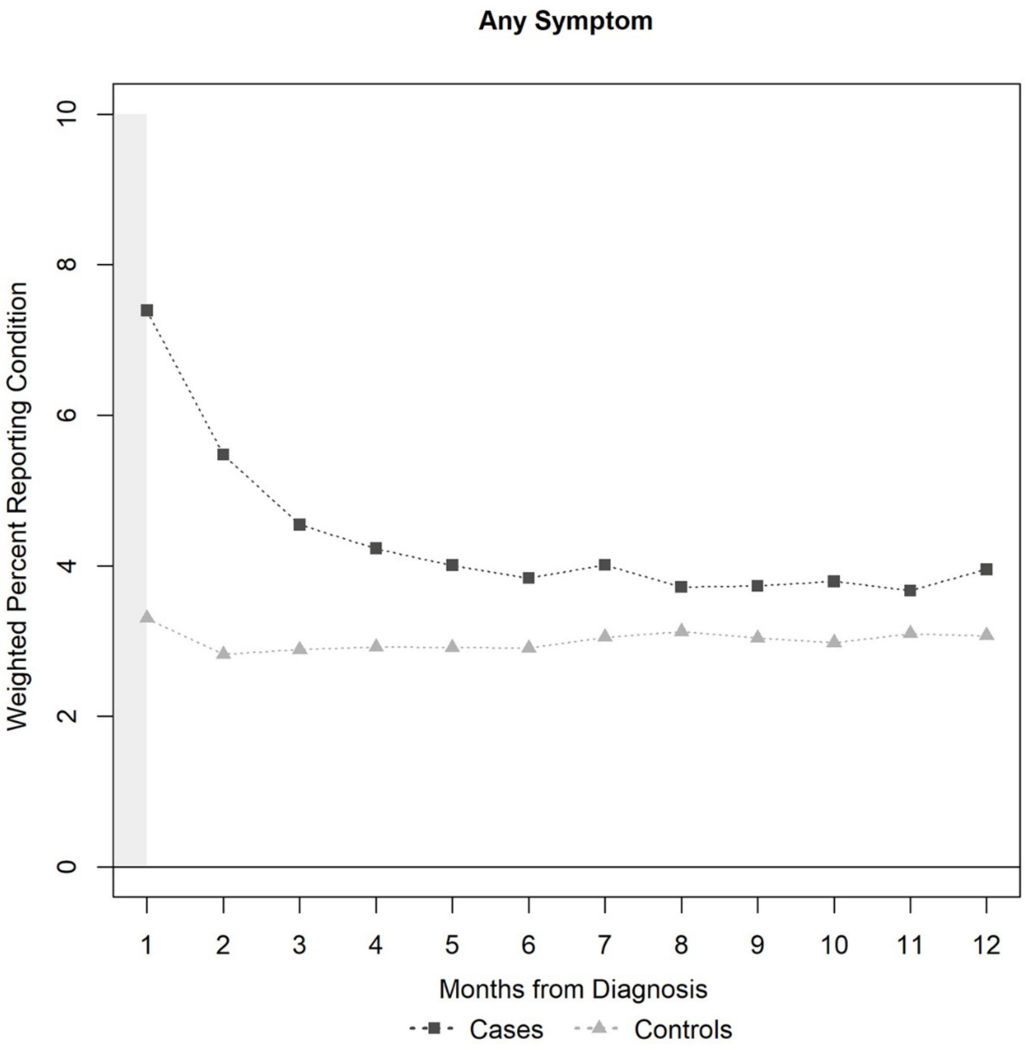

Appendix Figure 1. Weighted proportion of case-patients and control-patients with any nonspecific symptom code by month among case-patients with new onset symptoms (those who did not have a symptom code in that category at any point before the wash-out period) and matched control-patients in the year postdiagnosis, MarketScan database, 2017–2021.

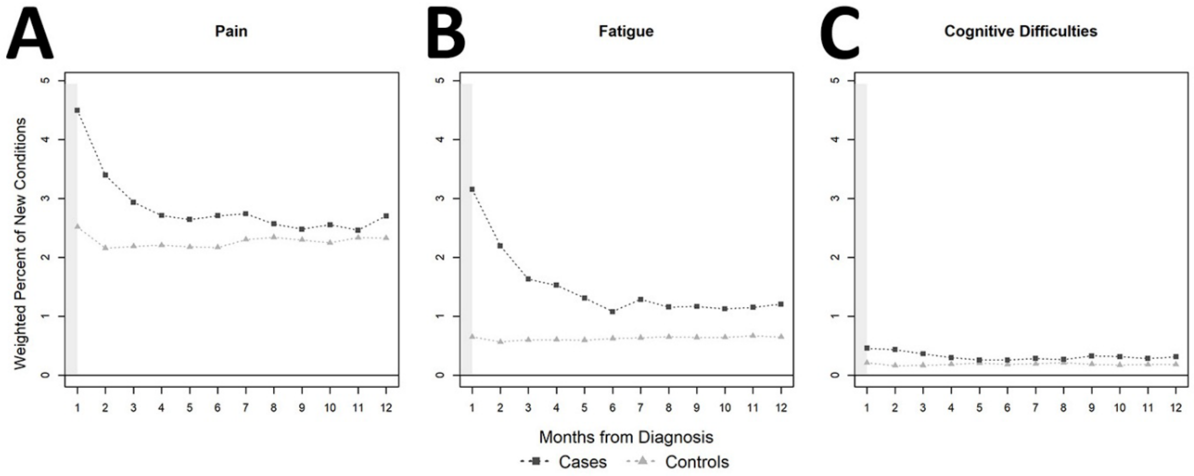

Appendix Figure 2. Weighted proportion of case-patients and control-patients with codes in each nonspecific symptom category by month among case-patients with new onset symptoms (those who did not have a symptom code in that category at any point before the wash-out period) and matched control-patients in the year postdiagnosis, MarketScan database, 2017–2021.

Reference

1. Zou GY, Donner A. Construction of confidence limits about effect measures: a general approach. Stat Med. 2008;27:1693–702. PubMed https://doi.org/10.1002/sim.3095
